# Dynamics of Donor-Derived Cell-Free DNA at the Early Phase After Pediatric Kidney Transplantation: A Prospective Cohort Study

**DOI:** 10.3389/fmed.2021.814517

**Published:** 2022-01-07

**Authors:** Weijian Nie, Xiaojun Su, Longshan Liu, Jun Li, Qian Fu, Xirui Li, Chenglin Wu, Jiali Wang, Ronghai Deng, E. Chen, Shicong Yang, Shujuan Li, Huanxi Zhang, Changxi Wang

**Affiliations:** ^1^Organ Transplant Center, The First Affiliated Hospital, Sun Yat-sen University, Guangzhou, China; ^2^Guangdong Provincial Key Laboratory on Organ Donation and Transplant Immunology, Guangzhou, China; ^3^Guangdong Provincial International Cooperation Base of Science and Technology (Organ Transplantation), Guangzhou, China; ^4^Department of Nephrology, The First Affiliated Hospital, Sun Yat-sen University, Guangzhou, China; ^5^Department of Pathology, The First Affiliated Hospital, Sun Yat-sen University, Guangzhou, China; ^6^Department of Cardiovascular Pediatrics, The First Affiliated Hospital, Sun Yat-sen University, Guangzhou, China

**Keywords:** pediatric kidney transplantation, donor-derived cell-free DNA, dynamics, donor-recipient size mismatch, pediatric donor

## Abstract

**Background:** Donor-derived cell-free DNA (ddcfDNA) has been suggested as an indicator of allograft injury in adult and pediatric kidney transplantation (KTx). However, the dynamics of ddcfDNA in pediatric KTx have not been investigated. In addition, it has not been demonstrated whether donor-recipient (D/R) size mismatch affect ddcfDNA level.

**Methods:** Pediatric KTx recipients with a single donor kidney were enrolled and followed up for 1 year. ddcfDNA, calculated as a fraction (%) in the recipient plasma, was examined longitudinally within 3 months post-transplant. D/R size mismatch degree was described as D/R height ratio. The 33rd percentile of D/R height ratio (0.70) was used as the cut-off to divide the patients into low donor-recipient height ratio group (<0.70) and high donor-recipient height ratio group (≥0.70). The dynamics of ddcfDNA were analyzed and the impact factors were explored. Stable ddcfDNA was defined as the first lowest ddcfDNA. ddcfDNA flare-up was defined as a remarkable elevation by a proportion of >30% from stable value with a peak value >1% during elevation.

**Results:** Twenty-one clinically stable recipients were enrolled. The median D/R height ratio was 0.83 (0.62–0.88). It took a median of 8 days for ddcfDNA to drop from day 1 and reach a stable value of 0.67% (0.46–0.73%). Nevertheless, 61.5% patients presented ddcfDNA>1% at day 30. Besides, 81.0% (17/21) of patients experienced elevated ddcfDNA and 47.6% (10/21) met the standard of ddcfDNA flare-up. Donor-recipient height ratio was an independent risk factor for ddcfDNA flare-up (odds ratio = 0.469 per 0.1, 95% CI 0.237–0.925, *p* = 0.029) and low donor-recipient height ratio (<0.70) was found to increase the risk of flare-up occurrence (odds ratio = 15.00, 95% CI 1.342–167.638, *p* = 0.028).

**Conclusions:** ddcfDNA rebounds in many stable pediatric KTx recipients without rejection. This may be induced by significant D/R size mismatch and may affect its diagnostic performance at the early phase after pediatric KTx in children.

## Introduction

Kidney transplantation (KTx) is the most effective treatment for children with end-stage renal disease (ESRD) (1, 2). However, long-term outcome of pediatric kidney transplantation is unsatisfactory and the 10-year graft survival rate is only about 60% (3). Antibody-mediated rejection (ABMR) is a crucial factor in poor long-term graft survival in both pediatric and adult kidney transplantation (4, 5). Despite increasing understanding of its pathogenesis, few sensitive and effective biomarkers are available for monitoring and diagnosis of ABMR. Renal allograft biopsy, though remaining the gold standard diagnosis of rejection, is infrequently used for surveillance because of the cost and its potential invasive complications (6). The conventional non-invasive parameters such as serum creatinine, cystatin C, and proteinuria are not sensitive and often lag renal allograft injury, leading to missed or delayed diagnosis of ABMR, and thus impact long-term graft outcome (7, 8). Accurate, reproducible, and timely non-invasive biomarkers to monitor graft injury and rejection are urgent to improve long-term transplant outcome.

Donor-derived cell-free DNA (ddcfDNA) has emerged as a potential and promising biomarker for early detection of graft injury and rejection. The ddcfDNA level above 1% strongly correlates with acute rejection (9). Accompanied with donor specific antibody (DSA), ddcfDNA could significantly improve the diagnosis efficiency of active ABMR (10). The utilization of ddcfDNA can better diagnose acute rejection and complement the Banff classification to treat subclinical rejection. High level of ddcfDNA has been proved to reveal subclinical graft injury caused by inadequate immunosuppression, while low level of ddcfDNA indicates absence of significant graft injury and thus facilitates to avoid unnecessary kidney biopsy (11). In patients with biopsy-proven early T cell-mediated rejection (TCMR), including Banff TCMR 1A and borderline lesions, ddcfDNA level could help risk stratification (12). The elevated ddcfDNA in those patients predicted adverse clinical outcomes including declined estimated glomerular filtration rate (eGFR), *de novo* DSA (*dn*DSA) formation and future rejection (12).

A high amount of ddcfDNA is released into peripheral circulation immediately after renal allograft reperfusion mainly due to ischemia-reperfusion injury (IRI), followed by a swift decrease to a stable low level (13). It's important to investigate the dynamics of ddcfDNA, especially in the early post-transplant period, as it is helpful to better interpret the clinical significance of ddcfDNA results, e.g., discrimination of IRI and rejection. Contrary to the accumulating data of ddcfDNA in adult KTx, only one study exploring ddcfDNA in pediatric KTx has been reported. Puliyanda et al. provided the evidence that ddcfDNA could be a useful indicator for identifying acute rejection in pediatric kidney transplantation (14). The dynamics of ddcfDNA in pediatric KTx, which may be different from that in adult KTx, has not been well-investigated. Many factors affect the level of ddcfDNA, such as infection and multiple organ transplantation. The fraction of ddcfDNA decreases in a recipient with infection mainly because of elevated white blood cell counts (15), while increases in a recipient with longer cold ischemia time (13, 16). It has not been demonstrated whether donor-recipient size mismatch affect ddcfDNA level. Pediatric kidney transplant program has developed fast during the past decades (5). Many pediatric donor kidneys are utilized for transplantation to pediatric recipients, which leads to variable donor-recipient (D/R) size mismatch (17–20). Since D/R size mismatch is considered to cause hyperfiltration injury (21), we hypothesized that the level of ddcfDNA, an indicator of allograft injury, may be affected by donor-recipient size mismatch. Therefore, the Pediatric kidney transplantation Cell-free DNA Trial (Pedi-ceft Study), was conducted to firstly demonstrate the dynamics of ddcfDNA in pediatric recipients early after kidney transplantation, and more importantly to explore its impact factors with especial attention to D/R size mismatch.

## Materials and Methods

### Study Design

This is a single-center prospective cohort study conducted in the First Affiliated Hospital of Sun Yat-sen University. Children who received primary pediatric deceased-donor-kidney transplantation between December 2019 and June 2020 were consecutively investigated. All enrolled pediatric recipients were scheduled to receive sequential ddcfDNA detection within 3 months and were followed up for 1 year. Patients with post-transplant complications within 3 months including graft loss, rejection, and polyomavirus-associated nephropathy were excluded. Patients with substandard specimen quality and those who were unable to finish 1-year follow-up were also excluded.

All patients and their legal representatives provided written informed consents for participation in the study. This study was approved by the institutional review board of the First Affiliated Hospital of Sun Yat-sen University and was registered at the official national Chinese Clinical Trial Registry (No. ChiCTR2000032333). This study was conducted in accordance with the principles of the World Medical Association Declaration of Helsinki and the declaration of Istanbul. No organs were from executed prisoners.

### ddcfDNA Measurements

Blood (8 mL) samples were drawn from enrolled patients using customized cfDNA blood collection tubes (Streck, Omaha, NE). Plasma was separated by centrifugation at 1,600×g for 10 min followed by a second centrifugation at 16,000×g for another 10 min. Plasma cfDNA was extracted with Circulating Nucleic Acid kit (Cat. No. 55114, Qiagen) and whole blood genomic DNA was extracted with DNA Blood mini kit (Cat. No. 51104, Qiagen). Purified DNA was quantified by Qubit 3.0 using the dsDNA HS Assay Kit (Life Technologies). Sequencing libraries were prepared by applying KAPA Hyper Prep kit (KAPA Biosystems) with 30 ng of input DNA. A total of 6,200 human single nucleotide polymorphism (SNP) loci were enriched by liquid hybridization based on SNP loci selection criteria (13). All sequencing was performed on the Illumina X-ten next generation sequencing (NGS) platform (10 ± 5 million, PE 150 bp). Sequencing data were processed as previously described (13). In brief, the SNP genotype of the recipient was determined by whole blood genomic DNA sequencing. Assuming that “a” is the donor genotype, the effective SNP sites that could be used for subsequent quantification including the recipient genotype, which was designated “AA,” and the donor genotype “Aa” or “aa,” were used to calculate the proportion distribution of donor SNP by the value of a/(A+a). According to the proportion of donor genotype “a” in the effective SNP, the fraction of ddcfDNA level was calculated based on Bayes approach.

### Parameters of ddcfDNA Dynamics

The ddcfDNA level was examined longitudinally and ddcfDNA fraction (%) was determined at day 1, day 4, day 7, day 14, day 30, day 60, and day 90, within an acceptable time window for each time point. Dynamic change of ddcfDNA was explored and depicted.

Stable ddcfDNA was defined as the first lowest ddcfDNA value after transplantation. Elevation of ddcfDNA was observed in a proportion of study subjects after reaching the stable level. Therefore, the dynamic characteristics of ddcfDNA elevation was especially described as the followings. The occurrence time of ddcfDNA elevation was presumed as the previous detection time point before its elevation, and therefore it was the same as the time point when ddcfDNA reached a stable level. The duration time of ddcfDNA elevation was counted as the period from occurrence of ddcfDNA elevation to its peak level. Peak ddcfDNA was the highest level of ddcfDNA during the elevation. The proportion of ddcfDNA change from stable level to peak level was calculated as (peak ddcfDNA – stable ddcfDNA)/stable ddcfDNA. Slight fluctuation in ddcfDNA was justified and considered as random intra-patient variations (11), whereas remarkable ddcfDNA elevation was considered abnormal and quantified using a parameter named FLARE-UP. Flare-up was defined when ddcfDNA elevated by over 30% from stable level as well as the peak level was over 1% during elevation. One percent was used as the threshold of flare-up because it was often used for the diagnosis of rejection and impaired renal allograft function in prior studies (14).

### Clinical Parameters and Clinical Events

The pre- and post-transplant demographics and clinical data of donors and recipients, including gender, age, height, weight, body surface area (BSA), human leukocyte antigen (HLA) mismatch, donor type, cold ischemia time (CIT), donor-recipient size mismatch, and immunosuppressive therapy were recorded. The size of child is often assessed by weight, height or BSA. BSA was calculated using Stevenson's BSA formula (22): *BSA*(*m*^2^) = 0.0061 × *height*(*cm*) + 0.0128 × *weight* (*kg*) −0.1529. In pediatric patients with kidney disease, height is preferred because ascites, edema or other complications can significantly affect weight. In this study, D/R size mismatch degree was described by the ratio of donor height to recipient height (23, 24). The 33rd percentile of D/R height ratio (0.70) was used as the cut-off to divide the patients into low D/R height ratio group (<0.70) and high D/R height ratio group (≥0.70).

Delayed graft function (DGF) was defined as the need for dialysis within the first week post-transplant. All recipients received tacrolimus as a part of the maintenance immunosuppression regimen. Tacrolimus trough levels were measured at least once a week in the first month, every 2–3 weeks in 2–3 months, every 4–6 weeks in 4–6 months, and every 1–2 months after 6 months. In addition, the frequency of detection was adjusted according to the clinical condition. The tacrolimus target trough level was 6.0–10.0 ng/mL in the first month, 5.0–9.0 ng/mL within 3 months, and 5.0–8.0 ng/mL thereafter. The estimated glomerular filtration rate (eGFR) calculated with Schwartz formula was used for assessment of kidney graft function (25, 26). Within 3 months post-transplant, eGFR was calculated and recorded weekly, and after that once per month. Graft ultrasound examination was longitudinally implemented during the follow-up, and the length of the kidney was measured to observe graft growth. Within the first 3 months post-transplant, at least one ultrasound examination was performed, and indicational ultrasound examination was performed after that. The proportion of graft growth was calculated as (kidney length after 3 months – kidney length within 3 months)/kidney length within 3 months.

All enrolled recipients were followed up for at least 1 year. Post-transplant complications including *dn*DSA, rejection and BK polyomavirus-associated nephropathy (BKVN) were prospectively recorded. After transplantation, DSA detection was conducted monthly within the first 3 months and every 3 months after that. Diagnosis of rejection and BKVN was confirmed by renal allograft biopsy following the 2018 Banff criteria (27). Patients with stable eGFR (eGFR increased by <15% compared to the previous eGFR), and without *dn*DSA, were considered clinically stable. Those with eGFR increased by over 15% in two-consecutive detection were advised to receive kidney biopsy. The ddcfDNA results were blinded to transplant physicians therefore no intervention would be exerted on patients with increased or abnormal ddcfDNA measurements.

### Statistical Analysis

Continuous data were presented as median (lower quartile-upper quartile) if not especially mentioned. Categorical data were reported as counts and percentages. Statistical analysis was performed using IBM SPSS Statistics version 22.0 (IBM Corporation, New York, USA), R version 4.0.3 (https://www.r-project.org) and GraphPad Prism 7 (GraphPad Software, La Jolla, CA). Comparisons between groups were performed using Mann-Whitney *U*-test for continuous variables and Chi-square test or Fisher's exact test for categorical variables. Spearman correlation test was used to test correlation between variables. Logistic regression was used for the univariable and multivariable analysis of risk factors for ddcfDNA flare-up and post-transplant outcomes. Linear regression was used for univariable and multivariable analysis of stable ddcfDNA, ddcfDNA at day 90, occurrence time and duration time of ddcfDNA elevation. The variables with *P* < 0.10 in the univariable analysis were enrolled in the multivariable model. Demographic and clinical characteristics of recipients and donors, including donor-recipient size mismatch degree, were included as the independent variables in the regression models. Dynamics parameters of ddcfDNA were included in the regression models for post-transplant outcomes. Adjusted *R* square and Somer's Delta were used for model performance evaluation. For all analyses, a *P* < 0.05 was considered statistically significant.

## Results

### Patient Demographics

**Figure 1:**
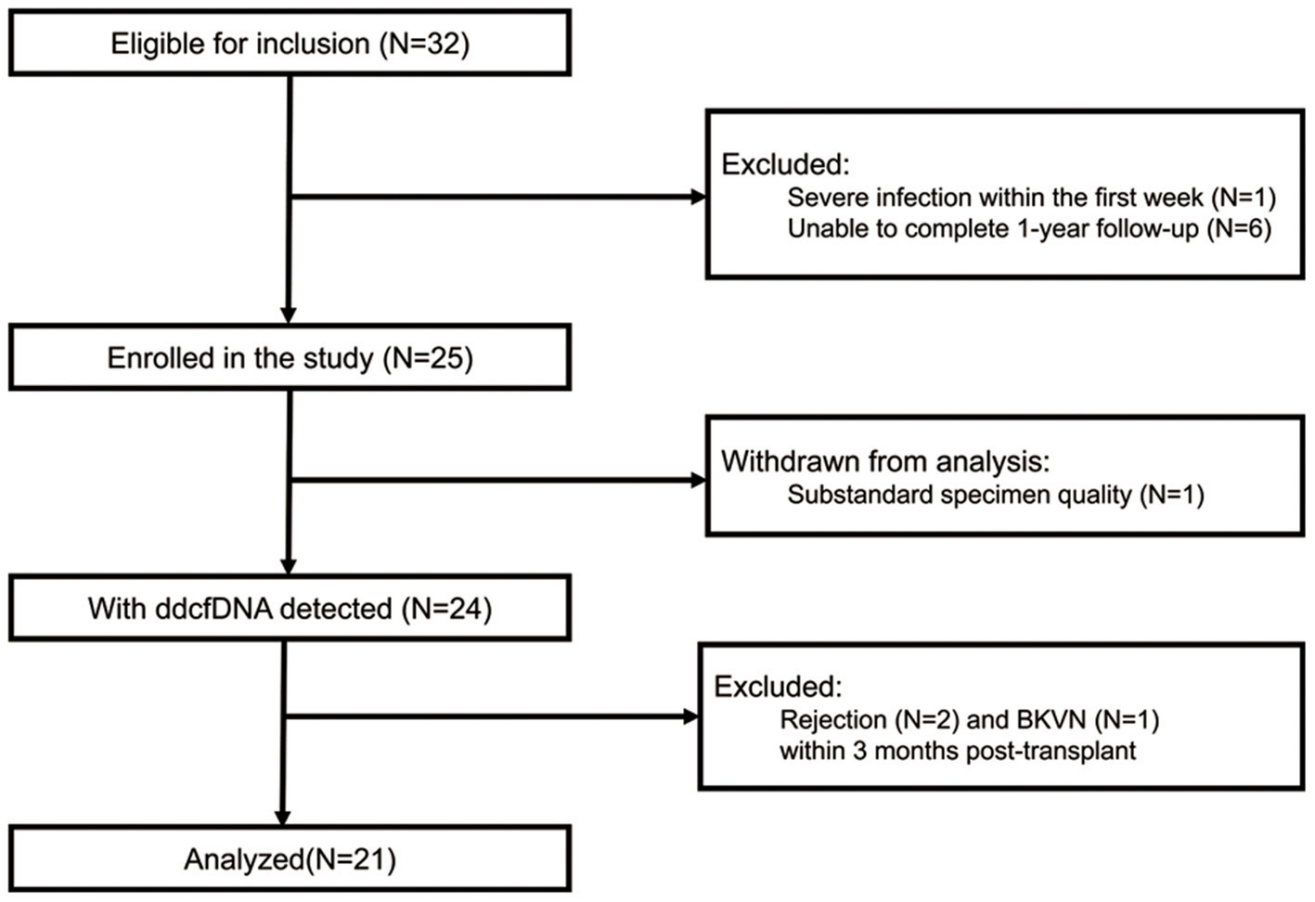
Flow diagram of recruitment and exclusion of study participants. BKVN, BK polyomavirus-associated nephropathy; ddcfDNA, donor-derived cell-free DNA.

The flow diagram of recruitment and exclusion of study participants was shown in Figure 1. A total of 32 recipients were eligible for inclusion in the study. According to the exclusion criteria, 10 patients were excluded while one patient was withdrawn from analysis because of substandard blood specimen quality. Thus, 21 recipients were eventually enrolled in the analysis. One recipient had pre-transplant panel reactive antibodies (HLA class I antibody 3%). No recipient showed pre-transplant DSA and was diagnosed with *dn*DSA within 3 months post-transplant. According to graft function and DSA status, all the 21 recipients were clinically stable. The demographic and clinical characteristics of donors and recipients were summarized in Table 1. The median donor-recipient height ratio was 0.83 (0.62–0.88). The median weight and height of donors were 15 (13–21) kg and 102 (90–120) cm, respectively, while the median weight and height of recipients were 31 (18.5–38.5) kg and 137 (115–150) cm. All kidneys were from deceased donors and eight (35.1%) of them were from donation after circulatory death (DCD). All recipients received tacrolimus + corticosteroid + mycophenolic acid (MPA) as maintenance. The median tacrolimus trough levels in 3 months post-transplant were within the window between 6.0 and 9.0 ng/ml (Supplementary Figure 1).

**Table 1:** Demographic and clinical characteristics.

	**All (*N* = 21)**	**Flare-up (*N* = 10)**	**Non-flare-up (*N* = 11)**	***P*-value**
**Recipient**				
Age (years old, IQR)	11.0 (7–13)	12 (9.3–13.8)	11 (5.5–11.5)	0.147
Male (*n*, %)	11 (52.4%)	7 (70.0%)	4 (36.4%)	0.198
Height (cm, IQR)	137 (115–150)	143 (132.8–150)	123 (110–150.5)	0.324
Weight (kg, IQR)	31 (18.5–38.5)	33.4 (25.5–39)	30 (17.1–37.3)	0.622
BSA (m^2^, IQR)	1.09 (0.76–1.24)	1.16 (1.00–1.22)	0.98 (0.73–1.27)	0.622
Blood type				0.119
O (*n*, %)	8 (38.1%)	3 (30.0%)	5 (45.5%)	
B (*n*, %)	8 (38.1%)	3 (30.0%)	5 (45.5%)	
A (*n*, %)	4 (19.0%)	4 (40%)	0%	
AB (*n*, %)	1 (4.8%)	0%	1 (9.1%)	
Pre-transplant transfusion (*n*, %)	10 (47.6%)	6 (60.0%)	4 (36.4%)	0.395
Dialysis				0.183
Yes (*n*, %)	16 (76.2%)	5 (50.0%)	9 (81.8%)	
No (*n*, %)	5 (23.8%)	5 (50.0%)	2 (18.2%)	
Dialysis time (years, IQR)	0.63 (0–1.37)	0.43 (0–1.12)	0.63 (0.33–2.09)	0.282
Pre-transplant PRA (*n*, %)	1 (4.8%)	0%	1 (9.1%)	1.000
Pre-transplant DSA (*n*, %)	0%	0%	0%	1.000
HLA mismatch				0.949
2 (*n*, %)	3 (14.3%)	2 (20.0%)	1 (9.1%)	
3 (*n*, %)	2 (9.5%)	1 (10.0%)	1 (9.1%)	
4 (*n*, %)	4 (19.0%)	2 (20.0%)	2 (18.2%)	
5 (*n*, %)	10 (47.6%)	4 (40.0%)	6 (45.5%)	
6 (*n*, %)	2 (9.5%)	1 (10.0%)	1 (9.1%)	
Primary disease				0.373
Hereditary renal disease (*n*, %)	8 (19.0%)	2 (20.0%)	6 (36.4%)	
CAKUT (*n*, %)	5 (23.8%)	3 (30.0%)	2 (18.2%)	
Glomerulonephritis (*n*, %)	1 (4.8%)	1 (10.0%)	0%	
Interstitial nephritis (*n*, %)	1 (4.8%)	1 (9.1%)	0%	
Lupus nephritis (*n*, %)	1 (4.8%)	1 (10.0%)	0%	
Unidentified (*n*, %)	5 (23.8%)	2 (20.0%)	3 (27.3%)	
**Donor**				
Age (years old, IQR)	3 (2–8)	3.5 (2–5)	3 (2–8)	0.915
Male (*n*, %)	16 (76.2%)	7 (70.0%)	9 (81.8%)	0.635
Height (cm, IQR)	102 (90–120)	90 (73.8–103)	120 (90.5–124)	0.096
Weight (kg, IQR)	15 (13–21)	14.5 (12.2–17.8)	15.0 (13.0–22.0)	0.596
BSA (m^2^, IQR)	0.65 (0.56–0.87)	0.60 (0.42–0.69)	0.75 (0.57–0.88)	0.180
Donor type				0.080
DBD (*n*, %)	13 (61.9%)	4 (40.0%)	9 (81.8%)	
DCD (*n*, %)	8 (35.1%)	6 (60.0%)	2 (18.2%)	
Cold ischemia time (hours, IQR)	9 (8–12.5)	10.5 (8.6–12.5)	8.5 (7.8–9)	0.215
**D/R size mismatch**				
D/R height ratio (IQR)	0.83 (0.62–0.88)	0.60 (0.58–0.82)	0.84 (0.78–1.05)	0.017
D/R weight ratio (IQR)	0.67 (0.48–0.93)	0.57 (0.43–0.70)	0.70 (0.54–0.93)	0.192
D/R BSA ratio (IQR)	0.72 (0.52–0.85)	0.51 (0.46–0.77)	0.83 (0.66–0.95)	0.024
**Induction therapy**				0.586
IL-2RA (*n*, %)	18 (85.7%)	8 (80.0%)	10 (90.9%)	
ATG (*n*, %)	3 (14.3%)	2 (20.0%)	1 (9.1%)	
**Maintenance therapy** (Tacrolimus + Corticosteroid + MPA)				0.063
MMF (*n*, %)	15 (71.4%)	5 (50.0%)	10 (90.9%)	
EC-MPS (*n*, %)	6 (28.6%)	5 (50.0%)	1 (9.09%)	
**DGF** (*n*, %)	3 (14.3%)	2 (20.0%)	1 (9.09%)	0.456

*ATG, anti-thymocyte globulin; BSA, body surface area; CAKUT, congenital anomalies of the kidney and urinary tract; DBD, donation after brain death; DCD, donation after circulatory death; DGF, delayed graft function; D/R, recipient-donor; DSA, donor specific antibody; EC-MPS, enteric-coated mycophenolate sodium; HLA, human leukocyte antigen; IL-2RA, Interleukin 2 receptor antibody; IQR, interquartile range; MMF, mycophenolate mofetil; MPA, mycophenolic acid; PRA, panel reactive antibodies*.

### ddcfDNA Dynamics

**Figure 2:**
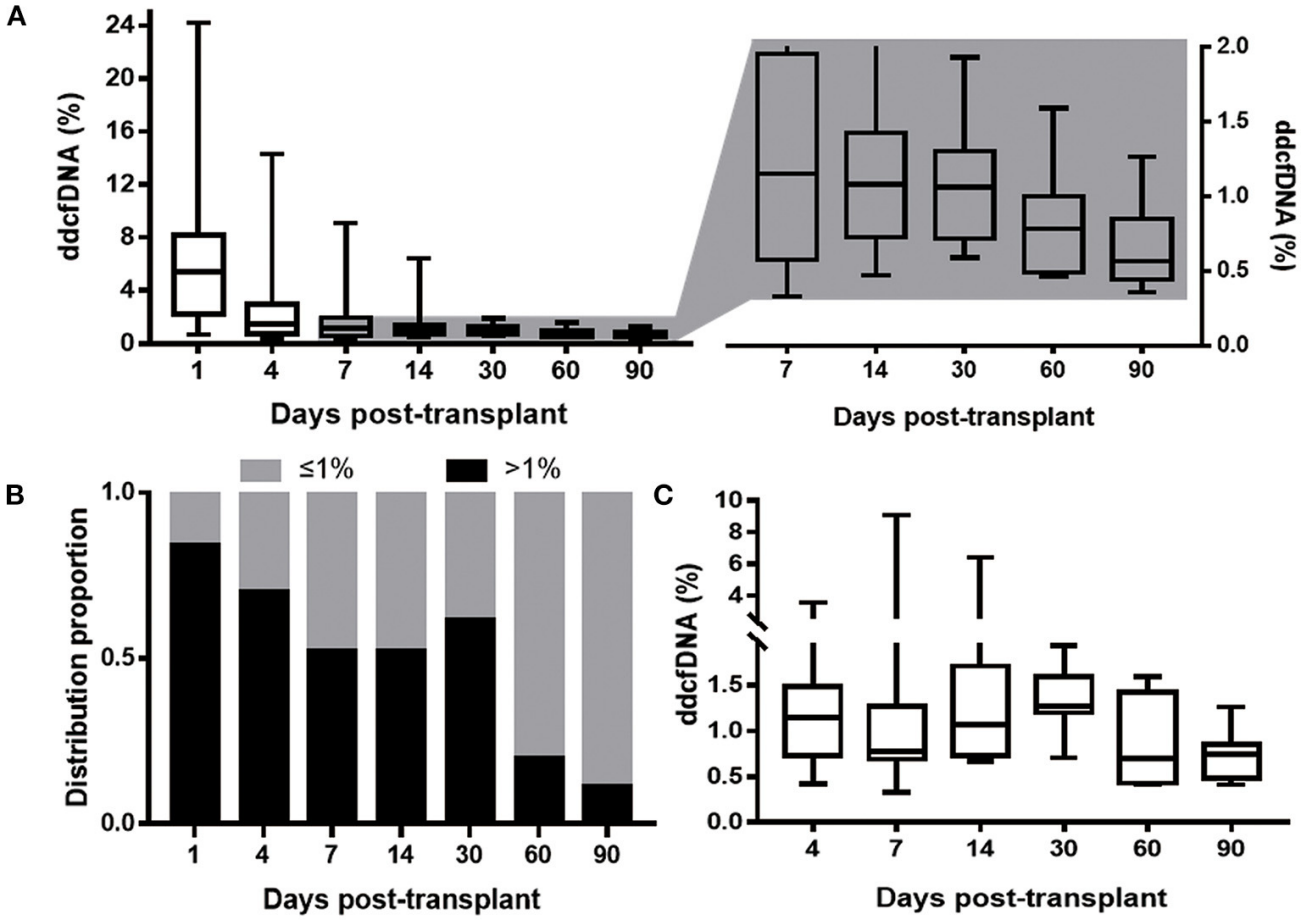
ddcfDNA at different time during the study period. **(A)** ddcfDNA change within 3 months. The boxes displaying ddcfDNA after day 4 was zoomed in and the elevation of ddcfDNA level could be indicated; **(B)** distribution proportion of 21 clinically stable patients with ddcfDNA >1% at different time post-transplant. The black bar depicts patients with ddcfDNA >1%; the gray bar depicts patients with ddcfDNA ≤ 1%; **(C)** ddcfDNA at different time (after day 1) in the ten patients with ddcfDNA flare-up. All the boxes depict the 25th and 75th percentiles as a box and a median line; whiskers extend to minimum or maximum. ddcfDNA, donor-derived cell-free DNA.

A total of 122 specimens of ddcfDNA were analyzed. All dynamic parameters were presented in Table 2. The median ddcfDNA was 5.4% at day 1, and subsequently decreased to stable level of 0.67% (0.46–0.73%) within a median of 8 days (Figure 2A). ddcfDNA in a high proportion of participants was higher than 1% within the first month post-transplant. The proportion of patients with ddcfDNA >1% at each time point was 84.2% at day 1, 66.7% at day 4, 57.1% at day 7, 52.4% at day 14, and 61.5% at day 30 (Figure 2B). This proportion remarkably decreased to 20.0% at day 60 and 11.1% at day 90.

**Table 2:** ddcfDNA dynamic parameters.

**Dynamic parameters**	**Analyzed patients (*N* = 21)**
**ddcfDNA at each time point**	
ddcfDNA at day 1 (%, IQR)	5.4% (2.25–7.89%)
ddcfDNA at day 4 (%, IQR)	1.49% (0.74–2.73%)
ddcfDNA at day 7 (%, IQR)	1.15% (0.61–1.76%)
ddcfDNA at day 14 (%, IQR)	1.08% (0.73–1.39%)
ddcfDNA at day 30 (%, IQR)	0.90% (0.71–0.85%)
ddcfDNA at day 60 (%, IQR)	0.64% (0.47–0.87%)
ddcfDNA at day 90 (%, IQR)	0.63% (0.46–0.86%)
Stable ddcfDNA (%, IQR)^a^	0.67% (0.46–0.73%)
ddcfDNA stable time (days, IQR)	8 (5–14)
Exact time of day 1 (hours, IQR)	26 (12–28)
**ddcfDNA elevation**	17 (81.0%)
Flare-up (*n*, %)^b^	10 (47.6%)
Elevation occurrence time post-operation (days, IQR)	6 (5–13)
Elevation duration (days, IQR)	10 (3–42)
Peak ddcfDNA during elevation (%, IQR)	1.27% (0.88–1.93%)
Proportion of ddcfDNA change during elevation (%, IQR)	80% (66–144%)

*ddcfDNA, donor-derived cell-free DNA; IQR, interquartile range*.

a *Stable ddcfDNA was defined as the first lowest ddcfDNA*.

b *Flare-up was defined as a post-stable elevation in ddcfDNA by over 30% from stable with a peak of over 1% during elevation*.

It was worth noting that ddcfDNA did not maintain constantly after reaching its stable level, and 81.0% (17/21) participants showed an elevation of ddcfDNA after stable time points (Figure 2). As for dynamic characteristics, ddcfDNA elevation occurred at day 6 (5–13 days), lasted for a median time of 10 days (3–42 days), and reached a peak level of 1.27% (0.88–1.93%). The proportion of ddcfDNA change from stable level to peak level during the elevation period was 80% (66–144%) in these 17 patients with ddcfDNA elevation. The elevation of ddcfDNA met the standard of flare-up in 47.6% (10/21) participants. ddcfDNA at different time points in these 10 patients with ddcfDNA flare-up was displayed in Figure 2C.

### Impact Factors of ddcfDNA Dynamics

Linear analysis in Table 3 demonstrated the potential factors affecting the stable ddcfDNA. An increase in donor weight, donor height, donor BSA, donor age, or female donor, was associated with elevated stable ddcfDNA in univariable analysis. However, the five parameters showed severe multicollinearity and thus were not appropriate for multiple regression. The adjusted R square values of the univariable linear models with donor weight, height, BSA, gender, and age were 0.382, 0.120, 0.255, 0.368, and 0.131 respectively, indicating that donor weight had the highest explainable level in the linear model (β coefficient = 0.017 per 1 kg, 95% CI 0.007–0.026, *P* = 0.002). The potential impact factors of ddcfDNA at day 90, the occurrence time and duration time of ddcfDNA elevation were summarized in Supplementary Table 1. Longer CIT and DCD donor was associated with increased ddcfDNA (%) at day 90. Due to the strong correlation between these two variables, it was unavailable to put them in to a multiple regression model. CIT had a higher explainable level in the model compared with donor type (adjusted *R* square, 0.358 vs. 0.311). Besides, DCD donor was found significantly correlated with the duration time of elevation (β coefficient = 35.352, CI: 13.317–57.386, *p* = 0.003).

**Table 3:** Univariable analysis of stable ddcfDNA.

**Univariable analysis**			
**Factors**	**β coefficient**	**95% CI**	***P*-value**
Recipient age (per year old)	0.021	−0.026 to 0.067	0.359
Recipient gender (male vs. female)	−0.086	−0.426 to 0.254	0.603
Recipient height (per 10 cm)	0.040	−0.038 to 0.117	0.295
Recipient weight (per 1 kg)	0.013	0.000 to 0.026	0.056
Recipient BSA (per 1 m^2^)	0.447	−0.106 to 1.000	0.107
Pre-transplant transfusion (yes vs. no)	0.069	−0.272 to 0.410	0.677
Pre-transplant dialysis (yes vs. no)	−0.059	−0.420 to 0.303	0.738
Pre-transplant dialysis time (per 1 year)	0.068	−0.050 to 0.185	0.241
Pre-transplant PRA (yes or no)	−0.305	−1.095 to 0.484	0.428
HLA mismatch (≥4 vs. <4)	0.067	−0.334 to 0.467	0.731
Donor age (per year old)	0.032	−0.003 to 0.067	0.069
Donor gender (male vs. female)	−0.529	−0.840 to −0.218	0.002
Donor height (per 10 cm)	0.048	−0.006 to 0.103	0.079
Donor weight (per 1 kg)^a^	0.017	0.007 to 0.026	0.002
Donor BSA (per 1 m^2^)	0.597	0.184 to 1.011	0.007
D/R height ratio (per 0.1)	0.039	−0.046 to 0.125	0.347
D/R weight ratio (per 0.1)	0.035	−0.012 to 0.082	0.136
D/R BSA ratio (per 0.1)	0.043	−0.020 to 0.106	0.168
DCD (yes vs. no)	0.005	−0.348 to 0.357	0.979
Cold ischemia time (per hour)	−0.020	−0.075 to 0.035	0.460
Induction therapy (ATG vs. IL-2RA)	0.262	−0.211 to 0.734	0.261
MPA type (EC-MPS vs. MMF)	0.176	−0.193 to 0.545	0.331
DGF (yes vs. no)	−0.133	−0.618 to 0.351	0.572

*ATG, anti-thymocyte globulin; BSA, body surface area; DCD, donation after circulatory death; DGF, delayed graft function; D/R, recipient-donor; DSA, donor specific antibody; EC-MPS, enteric-coated mycophenolate sodium; HLA, human leukocyte antigen; IL-2RA, Interleukin 2 receptor antibody; IQR, interquartile range; MMF, mycophenolate mofetil; MPA, mycophenolic acid; PRA, panel reactive antibodies*.

a *Only covariate of donor was enrolled in the final linear model because of the highest R square value*.

**Figure 3:**
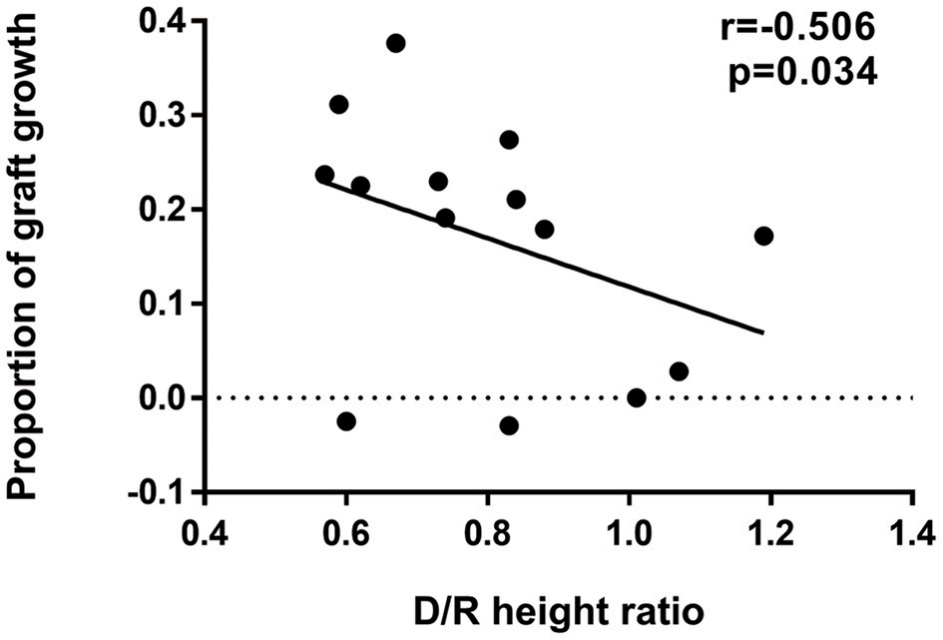
Correlation between D/R height ratio and proportion of graft growth. Negative correlation was observed between D/R height ratio (median of 0.79, IQR: 0.63–0.87) and proportion of graft growth (median of 20%, IQR: 6.4–23.5%). The proportion of graft growth was calculated as (kidney length after 3 months – kidney length within 3 months)/kidney length within 3 months and kidney lengths were determined by ultrasound examination. Correlation coefficient *r*, 95% CI of the coefficient *r* and *P*-value of the Spearman's correlation test was provided (*r* = −0.506, 95% CI: −0.823 to 0.051, *p* = 0.034). D/R, donor-recipient; CI, confidence interval.

The comparisons of flare-up and non-flare-up group were shown in Table 1. Donor-recipient height ratio was significantly lower in the flare-up group than that in non-flare-up group (Table 1, median 0.61 vs. 0.84, *p* = 0.017). Factors including D/R height ratio, donor height, donor type, MPA type, and D/R BSA ratio were potential risk factors (*P* < 0.10). D/R height ratio, donor type and MPA type were included in the multivariable analysis of flare-up while donor height and D/R BSA ratio were excluded due to the correlations with D/R height ratio. The potential risk factors on flare-up were investigated by univariable and multivariable analysis in Table 4. Model 2 showed that higher D/R height ratio was associated with lower risk of flare-up occurrence (odds ratio = 0.469 per 0.1, 95% CI: 0.237–0.0.925, *p* = 0.029). Recipients were divided into low D/R height ratio (<0.70) and high D/R height ratio group (≥0.70) according to the 33rd percentile of D/R height ratio (0.70). Model 1 indicated that low D/R height ratio increased the risk of flare-up occurrence (odds ratio = 15.00 low vs. high, 95% CI: 1.342–167.638, *P* = 0.028) compared with the high ratio group. The multivariable model 3 and 4 in Table 4 showed that after the adjustments, the effect of D/R height ratio on flare-up remained statistically significant, indicating that D/R height ratio was an independent risk factor for the occurrence of flare-up. The length of renal allograft gradually increased after transplantation, and the median proportion of increased kidney length (graft growth) was 20.0% (6.4–23.5%) after 3 months post-transplant. In addition, the proportion of graft growth was negatively correlated with donor-recipient height ratio (*r* = −0.506, *p* = 0.034, Figure 3).

**Table 4:** Univariable and multivariable analysis of flare-up.

**Univariable and multivariable analysis**				
	**(Adjusted) OR**	**95% CI**	***P*-value**	**Somer's Delta**
**Model 1**				0.509
D/R height ratio (low vs. high)^a^	15.00	1.342–167.638	0.028	
**Model 2**				0.618
D/R height ratio (per 0.1)	0.469	0.237–0.925	0.029	
**Model 3**				0.727
D/R height ratio (per 0.1)	0.493	0.243–0.997	0.049	
Donor type (DCD vs. DBD)	5.874	0.531–64.939	0.149	
**Model 4**				0.800
D/R height ratio (per 0.1)	0.246	0.061–0.992	0.049	
MPA type (EC-MPS vs. MMF)	115.656	0.956–13985.601	0.052	

*BSA, body surface area; CI, confidence interval; DBD, donation after brain death; DCD, donation after circulatory death; D/R, recipient-donor; EC-MPS, enteric-coated mycophenolate sodium; MMF, mycophenolate mofetil; MPA, mycophenolic acid; OR, odds ratio*.

a *The 33rd percentile of D/R height ratio (0.70) was used as the cut-off to divide the patients into low D/R height ratio group (<0.70) and high D/R height ratio group (≥0.70)*.

*Notation: Model 1 and 2 were univariable models; model 3 and 4 were multivariable models. The other factors with P < 0.10, including donor height and D/R BSA ratio were not enrolled for the adjustment as the result of collinearity with D/R height ratio*.

### Post-transplant Outcomes

Post-transplant outcomes of the enrolled patients were summarized in Supplementary Table 2. Three patients were diagnosed with biopsy-proven acute rejection after 3 months post-transplant, two of whom were accompanied with *dn*DSA. There was one patient diagnosed with *dn*DSA but with a stable renal function. The eGFR of the 21 patients were 80.0 (65.2–91.3) ml/min/1.73 m^2^ and 86.8 (61.4–102.9) ml/min/1.73 m^2^ at 3 months and 1 year, respectively (Supplementary Figure 2).

Supplementary Table 3 summarized the potential factors affecting post-transplant outcomes, including *dn*DSA formation, rejection and lower eGFR. No significant correlation was found between donor-recipient height ratio and any of the post-transplant outcomes. Moreover, no significant correlation was identified between the post-transplant outcomes and the dynamic parameters of ddcfDNA, including the stable ddcfDNA level, the occurrence and duration time of ddcfDNA elevation as well as ddcfDNA flare-up.

In addition, within 3 months after kidney transplantation, there was no pathogen-proven infection and there was no any sign of recurrence of the primary disease in any recipient. No recipient experienced recurrence of primary diseases after 3 months. However, after 3 months post-transplant, three recipients were diagnosed with infection (one case of cytomegalovirus pneumonia, one case of herpetic stomatitis, one case of BKVN).

## Discussion

This is the first study to investigate the dynamic characteristics of ddcfDNA in pediatric kidney transplantation and to explore the impact factors of ddcfDNA dynamics. We observed a rapid decline of ddcfDNA level within 1 week after its initial increase post-transplant and reached a stable level <1%. Nevertheless, ddcfDNA fluctuated and presented an abnormal elevation defined as ddcfDNA flare-up after the first week post-transplant. Further analysis revealed that ddcfDNA flare-up was correlated with donor-recipient size mismatch. This study depicted the early change of ddcfDNA in pediatric KTx recipients and facilitates interpretation in its clinical application.

In this cohort of pediatric recipients, high-level ddcfDNA at day 1 rapidly declined and reached a stable level <1%. This dynamic characteristic was similar as the change pattern of ddcfDNA in adult KTx recipients (13), while there were subtle differences. Firstly, it took a longer time to reach a stable level which could be explained by more deceased donor kidneys in this study, since the adult cohort included KTx from living-related donors (13). Secondly, the stable level of ddcfDNA was lower as 0.67% in this study. One possible explanation is all donor kidneys were from deceased pediatric donors, since a higher stable ddcfDNA level was observed in participants with larger donor-weight kidneys (Table 3). Thirdly, an abnormal elevation of ddcfDNA was observed after it reached a stable level, and this significant but not subtle elevation was not observed in the adult cohorts (13, 28).

Puliyanda et al. suggested the ddcfDNA level of 1% as the cut-off value for ABMR diagnosis in pediatric KTx recipients, which was the same in adult KTx studies (9, 14). Unexpectedly, over half of stable pediatric recipients in this study presented a ddcfDNA level >1% at day 14 and day 30 after transplantation, and ddcfDNA was over 1% in 11.1% of participants at 3 months post-transplant. These results suggest the cut-off value of 1% would significantly lead to a high false positive probability when ddcfDNA is applied for ABMR diagnosis at the early phase after kidney transplantation in children. On the other hand, we did observe a very high level of ddcfDNA (>4%) in two children with biopsy-proven ABMR, respectively, at day 70 and day 30 post-transplant (8.42 and 4.18%, Supplementary Figure 3). Therefore, we suggest careful interpretation of ddcfDNA >1% in children early after KTx, while remaining cautious about a very high-level ddcfDNA to timely diagnosis and treatment of ABMR.

Subtle elevation of ddcfDNA was observed after reaching the stable level both in our pediatric KTx recipients and adult KTx recipients (13, 28), and such elevation was considered acceptable in clinically stable patients (11). However, we found a significant abnormal elevation pattern of ddcfDNA within 3 months post-transplant, which was inconsistent with graft function and *dn*DSA. Such abnormal elevation pattern was denoted as flare-up. We found that pediatric recipients with donor-recipient size mismatch were at high risk of ddcfDNA flare-up (Table 4). Severe donor-recipient size mismatch is harmful and is considered to induce graft injury (29, 30). ddcfDNA flare-up might be a result of hyperfiltration graft injury induced by donor-recipient size mismatch. Donor-recipient size mismatch was reported to increase kidney graft loss in adolescents (30, 31). Donor-recipient size mismatch led to rapid adaptive growth of pediatric donor kidneys (20), and the hyperfiltration injury might alleviate during this compensatory process, since the elevated ddcfDNA did not continuously increase and instead tended to decrease to a low level. In this study, no significant correlation was found between D/R size mismatch and the post-transplant outcomes including graft survival.

This study has the limitations of relatively small cohort size and short follow-up period. We were unable to assess the potential impact of the early abnormal ddcfDNA on long-term graft outcome. Besides, longitudinal examination of ddcfDNA with more frequent time points would facilitate full comprehension of ddcfDNA change patterns in pediatric KTx.

In summary, this study firstly demonstrated the dynamic characteristics of ddcfDNA after pediatric kidney transplantation. A high proportion of participants presented ddcfDNA >1% at day 30 post-transplant. A significant rebound (flare-up) of ddcfDNA was observed, and this may reflect the hyperfiltration injury of grafts caused by donor-recipient size mismatch. The study results suggest one should carefully interpret the clinical implication of ddcfDNA level at the early phase after pediatric kidney transplantation.

## Data Availability Statement

The data presented in the study are deposited in the NCBI Sequence Read Archive, accession number PRJNA789391.

## Ethics Statement

The studies involving human participants were reviewed and approved by the Institutional Review Board of The First Affiliated Hospital of Sun Yat-sen University. Written informed consent to participate in this study was provided by the participants' legal guardian/next of kin.

## Author Contributions

WN, XS, LL, HZ, and CWa: study design, data interpretation, and manuscript preparation. LL, JL, QF, CWu, RD, and SL: patient enrollment and informed consent. WN and XL: laboratory examination. WN and HZ: data analysis. WN, XS, JW, and EC: clinical data collection. SY: interpretation of pathological biopsies. XS, LL, HZ, and CWa: funding support, study supervision, and critical review of manuscript. HZ and CWa were responsible for the decision to submit for publication. All authors contributed to the article and approved the submitted version.

## Funding

This study was supported by Science and Technology Planning Project of Guangdong Province, China (2015B020226002 and 2017A020215012), National Natural Science Foundation of China (81870511), Key Scientific and Technological Program of Guangzhou City (201803040011), Guangdong Basic and Applied Basic Research Foundation (2020A1515010884), Guangdong Natural Science Foundation (2018A030313016), Guangdong Provincial Key Laboratory on Organ Donation and Transplant Immunology (2017B030314018 and 2020B1212060026), and Guangdong Provincial International Cooperation Base of Science and Technology (Organ Transplantation, 2020A0505020003).

## Conflict of Interest

The authors declare that the research was conducted in the absence of any commercial or financial relationships that could be construed as a potential conflict of interest. The reviewer BN declared a past co-authorship with several of the authors XS, LL, JL, QF, CWu, RD, HZ, and CWa to the handling editor.

## Publisher's Note

All claims expressed in this article are solely those of the authors and do not necessarily represent those of their affiliated organizations, or those of the publisher, the editors and the reviewers. Any product that may be evaluated in this article, or claim that may be made by its manufacturer, is not guaranteed or endorsed by the publisher.

## Supplementary Material

Supplementary Figure 1 Tacrolimus trough levels at different time post-transplant in all 21 recipients. Median tacrolimus trough levels (ng/ml) at 1 week, 2 weeks, 1 month, 3 months, 6 months, 1 year was 6.7 (5.7–8.6), 7.9 (6.9–9.3), 7 (6.0–8.0), 7.1 (6.2–8.2), 7.7 (6.7–8.6), and 6.3 (5.9–7.0). Boxes depict the 25th and 75th percentiles as a box and a median line; whiskers extend to minimum or maximum. Tac, tacrolimus; wk(s), week(s); mth(s), month(s); yr, year.

Supplementary Figure 2 The eGFR change at different time post-transplant in all 21 recipients. Median eGFR (ml/min/1.73m^2^) at 1 week, 2 weeks, 1 month, 3 months, 6 months, 1 year was 53.87 (40.49–77.19), 63.25 (49.27–82.15), 69.47 (53.13–74.46), 80.06 (65.18–91.25), 85.99 (69.28–102.23), and 86.78 (61.36–102.82). Boxes depict the 25th and 75th percentiles as a box and a median line; whiskers extend to minimum or maximum. Wk(s), week(s); mth(s), month(s); yr, year.

Supplementary Figure 3 ddcfDNA and clinical events in 2 children with rejection occurrence within 3 months. **(A,B)** Two children experienced worsen graft function, who were diagnosed with rejection within 3 months, and were excluded from the study. The white arrow and black arrow depict the occurrence time point of *de novo* DSA and rejection respectively. DSA, donor-specific antibody; ddcfDNA, donor-derived cell-free DNA.

Supplementary Table 1 Univariable analysis of other dynamics parameters.

Supplementary Table 2 Post-transplant outcomes within 1 year.

Supplementary Table 3 Odds ratio of size mismatch degree and dynamics parameters on post-transplant outcomes via univariable analysis.

## Abbreviations

ABMR: antibody-mediated rejection
ATG: anti-thymocyte globulin
BKVN: BK polyomavirus-associated nephropathy
BSA: body surface area
CAKUT: congenital anomalies of the kidney and urinary tract
CI: confidence interval
CIT: cold ischemia time
DBD: donation after brain death
DCD: donation after circulatory death
ddcfDNA: donor-derived cell-free DNA
DGF: delayed graft function
*dn*DSA: *de novo* donor specific antibody
D/R height ratio or size mismatch: donor-recipient height ratio or size mismatch
EC-MPS: enteric-coated mycophenolate sodium
eGFR: estimated glomerular filtration rate
ESRD: end-stage renal disease
HLA: human leukocyte antigen
IL-2RA: interleukin 2 receptor antibody
IQR: interquartile range
IRI: ischemia-reperfusion injury
KTx: kidney transplantation
MMF: mycophenolate mofetil
MPA: mycophenolic acid
NGS: next generation sequencing
OR: odds ratio
PRA: panel reactive antibodies
SNP: single nucleotide polymorphism
TCMR: T cell-mediated rejection.
