# Supplementary figures and images for: Dynamics of Donor-Derived Cell-Free DNA at the Early Phase After Pediatric Kidney Transplantation: A Prospective Cohort Study
Source: Front Med (Lausanne). 2022 Jan 7;8:814517. doi: 10.3389/fmed.2021.814517 (PMC8777035; doi:10.3389/fmed.2021.814517)

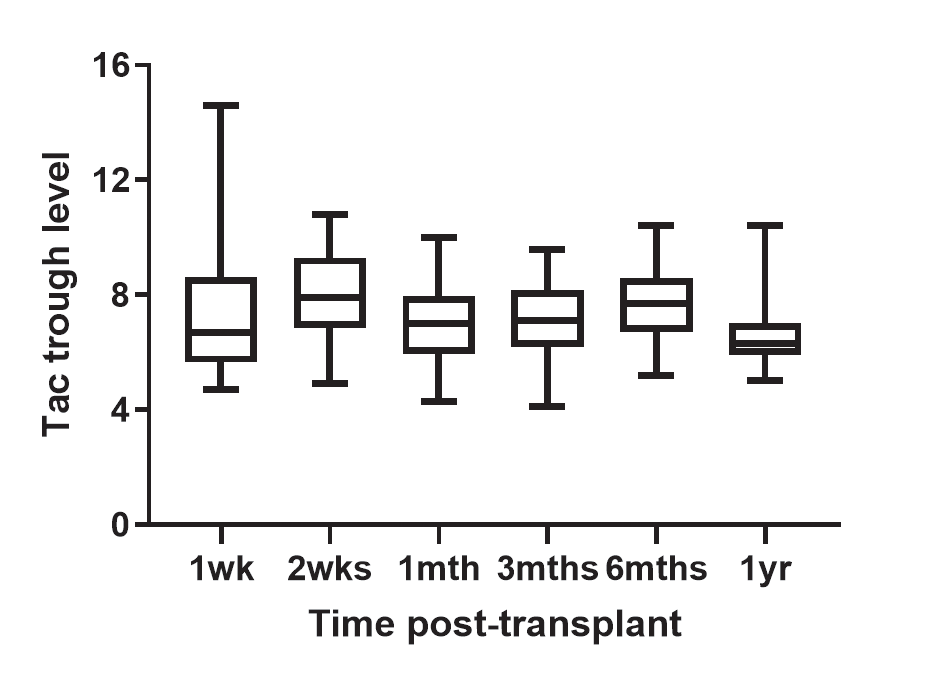

Supplement: Supplementary Figure 1 — Tacrolimus trough levels at different time post-transplant in all 21 recipients. Median tacrolimus trough levels (ng/ml) at 1 week, 2 weeks, 1 month, 3 months, 6 months, 1 year was 6.7 (5.7–8.6), 7.9 (6.9–9.3), 7 (6.0–8.0), 7.1 (6.2–8.2), 7.7 (6.7–8.6), and 6.3 (5.9–7.0). Boxes depict the 25th and 75th percentiles as a box and a median line; whiskers extend to minimum or maximum. Tac, tacrolimus; wk(s), week(s); mth(s), month(s); yr, year. [file Image_1.TIF]

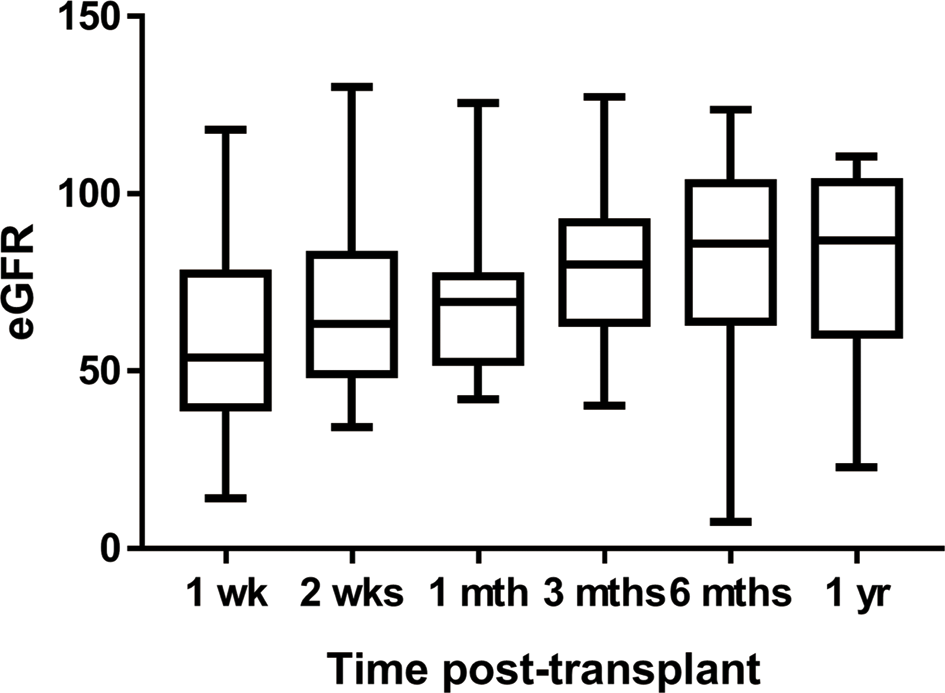

Supplement: Supplementary Figure 2 — The eGFR change at different time post-transplant in all 21 recipients. Median eGFR (ml/min/1.73m2) at 1 week, 2 weeks, 1 month, 3 months, 6 months, 1 year was 53.87 (40.49–77.19), 63.25 (49.27–82.15), 69.47 (53.13–74.46), 80.06 (65.18–91.25), 85.99 (69.28–102.23), and 86.78 (61.36–102.82). Boxes depict the 25th and 75th percentiles as a box and a median line; whiskers extend to minimum or maximum. Wk(s), week(s); mth(s), month(s); yr, year. [file Image_2.TIF]

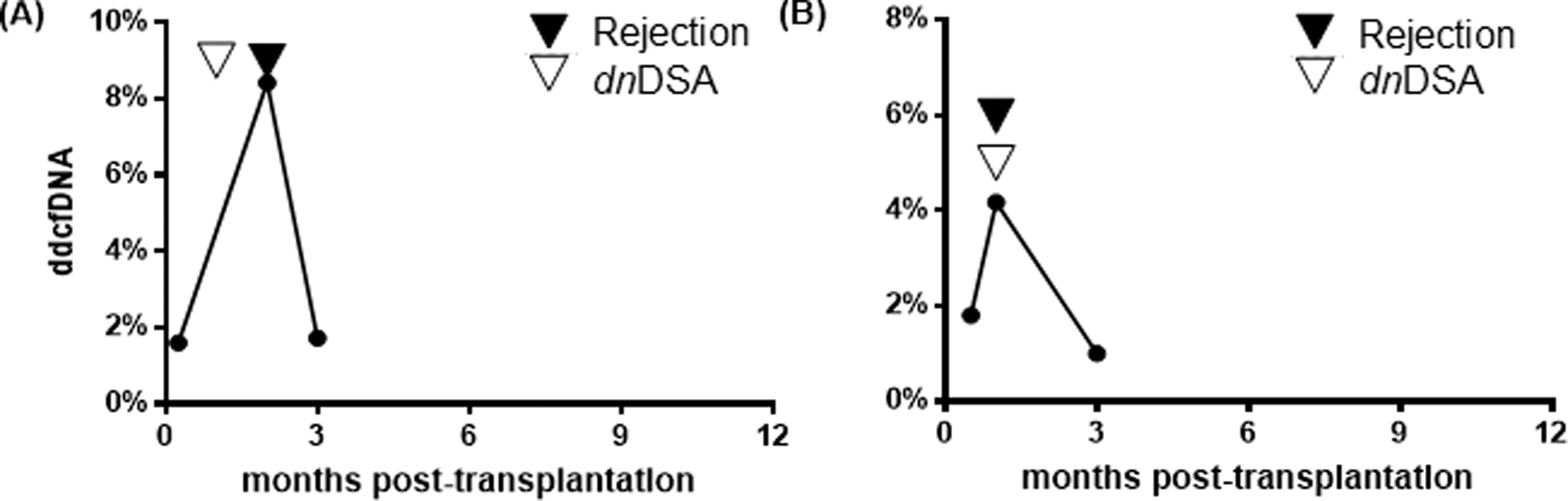

Supplement: Supplementary Figure 3 — ddcfDNA and clinical events in 2 children with rejection occurrence within 3 months. (A,B) Two children experienced worsen graft function, who were diagnosed with rejection within 3 months, and were excluded from the study. The white arrow and black arrow depict the occurrence time point of de novo DSA and rejection respectively. DSA, donor-specific antibody; ddcfDNA, donor-derived cell-free DNA. [file Image_3.TIF]
