# Supplementary material for: Dynamics of Donor-Derived Cell-Free DNA at the Early Phase After Pediatric Kidney Transplantation: A Prospective Cohort Study
Source: Front Med (Lausanne). 2022 Jan 7;8:814517. doi: 10.3389/fmed.2021.814517 (PMC8777035; doi:10.3389/fmed.2021.814517)
Supplement: Supplementary Table 1 — Univariable analysis of other dynamics parameters. [file Data_Sheet_1.docx]

| Supplemental table 1. Univariable analysis of other dynamics parameters | | | | |
| --- | --- | --- | --- | --- |
| Univariable analysis | | | | |
| Parameters | Factors^a^ | β coefficient | 95% CI | P-value |
| ddcfDNA at day 90 (%)^b^ | Cold ischemia time (per hour) | 0.050 | 0.017-0.082 | 0.005 |
|  | DCD (yes vs no) | 0.300 | 0.084-0.515 | 0.009 |
|  | D/R height ratio (per 0.1) | -0.009 | -0.075-0.056 | 0.703 |
| ddcfDNA elevation occurrence time (days) | D/R height ratio (per 0.1) | 25.407 | -11.133-61.947 | 0.159 |
| ddcfDNA elevation duration (days) | DCD (yes vs no) | 35.352 | 13.317-57.386 | 0.003 |
|  | D/R height ratio (per 0.1) | -2.859 | -69.961-64.243 | 0.930 |
| Abbreviations: CI, confidence interval; DCD, donation after circulatory death; D/R, donor-recipient.  ^a^ Only factor of D/R height ratio and factor with p value < 0.05 was displayed;  ^b^ ddcfDNA at day 90 was collected in 18 patients. | | | | |

| Supplemental table 2. Post-transplant outcomes within 1 year | |
| --- | --- |
| All (N=21) | |
| **Clinical events ^a^** |  |
| *De novo* DSA (n, %) | 3 (14.3%) |
| Rejection (n, %) | 3 (14.3%) |
| BKVN (n, %) | 1 (4.8%) |
| **Graft function** |  |
| eGFR at 3 months (ml/min/1.73m^2^, IQR) | 80.0 (65.2-91.3) |
| eGFR at 1 year (ml/min/1.73m^2^, IQR) | 86.78 (61.4-102.9) |
| eGFR < 60 at 1 year (n, %) | 5 (23.8%) |
| Abbreviations: BKVN, BK polyomavirus-associated nephropathy; DSA, donor specific antibody; eGFR, estimated glomerular filtration rate.  ^a^ Clinical events after the first 3 months post-transplant; | |

| Supplemental table 3. Odds ratio of size mismatch degree and dynamics parameters on post-transplant outcomes via univariable analysis | | | | | | | | | | | | |
| --- | --- | --- | --- | --- | --- | --- | --- | --- | --- | --- | --- | --- |
| All (n=21) | | | | | | | | | | | | |
| Factors | | *De novo* DSA | | | | Rejection | | | | eGFR < 60 | | |
|  | | OR | 95% CI | P-value | OR | | 95% CI | P-value | OR | | 95% CI | P-value |
| D/R size mismatch | D/R height ratio (per 0.1) | 1.345 | 0.700-2.584 | 0.373 | 1.534 | | 0.764-3.079 | 0.229 | 1.177 | | 0.720-1.921 | 0.516 |
| ddcfDNA dynamic parameters | Stable ddcfDNA (per 1%)^a^ | 0.388 | 0.006-27.336 | 0.663 | 1.185 | | 0.043-32.863 | 0.920 | 0.885 | | 0.060-13.142 | 0.929 |
|  | Flare-up (yes vs no)^b^ | 0.500 | 0.038-6.547 | 0.597 | 2.500 | | 0.191-32.802 | 0.485 | 1.143 | | 0.172-7.601 | 0.890 |
|  | Occurrence time of  ddcfDNA elevation (per 1 day) | 0.999 | 0.916-1.090 | 0.990 | 1.005 | | 0.928-1.089 | 0.906 | 0.971 | | 0.850-1.109 | 0.664 |
|  | Duration of ddcfDNA elevation (per 1 day) | 1.038 | 0.987-1.092 | 0.144 | 16.626 | | - | 0.992 | 0.997 | | 0.959-1.036 | 0.875 |
| Abbreviations: CI, confidence interval; ddcfDNA, donor-derived cell-free DNA; D/R, donor-recipient; DSA, donor specific antibody; eGFR, estimated glomerular filtration rate; OR, odds ratio.  ^a^ Stable ddcfDNA was defined as the first lowest ddcfDNA;  ^b^ Flare-up was defined as a post-stable elevation in ddcfDNA by over 30% from stable with a peak of over 1% during elevation. | | | | | | | | | | | | |
